# Activation of Toll-like Receptor 2 (TLR2) induces Interleukin-6 trans-signaling

**DOI:** 10.1038/s41598-019-43617-5

**Published:** 2019-05-13

**Authors:** Charlotte M. Flynn, Yvonne Garbers, Juliane Lokau, Daniela Wesch, Dominik M. Schulte, Matthias Laudes, Wolfgang Lieb, Samadhi Aparicio-Siegmund, Christoph Garbers

**Affiliations:** 1 Institute of Biochemistry, Kiel University, Kiel, Germany; 2 Institute of Psychology, Kiel University, Kiel, Germany; 3 Institute of Immunology, University Hospital Schleswig-Holstein, Campus Kiel, Kiel, Germany; 4 Department of Internal Medicine 1, Kiel University, Kiel, Germany; 5 Institute of Epidemiology, Kiel University, Kiel, Germany; 6 Present Address: Department of Pathology, Otto-von-Guericke-University Magdeburg, Medical Faculty, Magdeburg, Germany

**Keywords:** Interleukins, Interleukins, Signal transduction

## Abstract

Signaling of the pleiotropic cytokine Interleukin-6 (IL-6) via its soluble IL-6R (sIL-6R) has been termed trans-signaling and is thought to be responsible for the pro-inflammatory properties of IL-6. The sIL-6R can be generated by alternative mRNA splicing or proteolytic cleavage of the membrane-bound IL-6R. However, which stimuli induce sIL-6R release and which endogenous signaling pathways are required for this process is poorly understood. Here, we show that activation of Toll-like receptor 2 (TLR2) on primary human peripheral blood mononuclear cells (PBMCs) and on the monocytic cell line THP-1 induces expression and secretion of IL-6 and the generation of sIL-6R. We show by flow cytometry that monocytes are a PBMC subset that expresses TLR2 in conjunction with the IL-6R and are the major cellular source for both IL-6 and sIL-6R. Mechanistically, we find that the metalloproteases ADAM10 and ADAM17 are responsible for cleavage of the IL-6R and therefore sIL-6R generation. Finally, we identify the Extracellular-signal Regulated Kinase (ERK) cascade as a critical pathway that differentially regulates both IL-6 and sIL-6R generation in monocytes.

## Introduction

The cytokine Interleukin-6 (IL-6) is involved in numerous physiological and pathophysiological processes^1^. While IL-6 in healthy individuals is usually not detectable, IL-6 serum levels rise dramatically in all inflammatory conditions, with µg/ml during meningococcal septic shock as the most severe example^2^. Consequently, therapeutic compounds have been developed that block IL-6 activity, and the monoclonal antibody tocilizumab which blocks binding of IL-6 to its receptor (IL-6R) has been approved for the treatment of rheumatoid arthritis^3–5^.

IL-6 belongs to the IL-6 family of cytokines, whose hallmark is the signaling through the ubiquitously expressed glycoprotein 130 (gp130)^6^. However, IL-6 cannot directly activate gp130, but has to initially bind to a non-signaling membrane-bound IL-6R, a pathway that has been termed classic signaling^7^. This IL-6/IL-6R complex is responsible for the recruitment and subsequent formation of a gp130 homodimer, which leads to the activation of intracellular signaling cascades, e.g. the Jak/STAT pathway^6^. Of note, only few cell types, including hepatocytes and several leukocyte subsets, express the IL-6R on their cell surface, and the majority of cell types therefore is irresponsive to IL-6 classic signaling. However, soluble forms of the IL-6R (sIL-6R) do exist, which bind IL-6 with similar affinity as the membrane-bound IL-6R, and the IL-6/sIL-6R complex can induce formation of a gp130 homodimer on virtually all cell types, irrespective whether they express the IL-6R on their own^8^. This pathway has been termed IL-6 trans-signaling, and there is good evidence that it is responsible for the pro-inflammatory properties of IL-6, whereas IL-6 classic signaling accounts for the regenerative, anti-inflammatory properties of IL-6^7,9^. Recently, cluster signaling as a third pathway of IL-6 has been described, which is important for the generation of pathogenic Th17 cells^10^.

The sIL-6R can be generated by two distinct mechanisms: alternative splicing of the IL6R mRNA and proteolytic cleavage of the membrane-bound IL-6R. We have recently shown that only around 15% of the 40–80 ng/ml sIL-6R in human serum is generated by alternative splicing, whereas the majority of sIL-6R *in vivo* is generated by proteolytic cleavage^11^. The two metalloproteases ADAM10 and ADAM17 have been described to be responsible for sIL-6R generation^12–15^, and the cleavage sites of both proteases match the one identified *in vivo*^11^. Furthermore, sIL-6R serum levels are genetically controlled by the single nucleotide polymorphism (SNP) rs2228145, which results in the exchange of the amino acid residue Asp-358 to Ala-358 and increased sIL-6R serum levels^16,17^. Mechanistically, the Asp358Ala variation increases the susceptibility of the IL-6R towards proteolytic cleavage by ADAM10 and ADAM17^11,18^.

Surprisingly, endogenous stimuli and the responsible signaling pathways that induce sIL-6R release *in vivo* are poorly understood, because most *in vitro* studies use rather artificial activators of ADAM17 like the phorbol ester phorbol-12-myristate-13-acetate (PMA). Recently, injection of lipopolysaccharide (LPS) into mice, which represents an established endotoxemia model, has been shown to increase sIL-6R serum levels in an ADAM17-dependent manner^19^. Furthermore, LPS-induced ADAM17 activation has been shown to result in the release of other ADAM17 substrates, e.g. TNFα^20,21^.

LPS is a pathogen associated molecular pattern (PAMP) and forms part of the outer membrane of gram-negative bacteria. It activates Toll-like receptor 4 (TLR4), which belongs to the TLR family of pattern recognition receptors and is expressed mainly on antigen-presenting cells^22^. TLRs in humans comprise ten different type I-transmembrane proteins, which recognize a variety of pathogenic structures^23^. Activation of TLRs results in the activation of a number of intracellular signaling pathways, including the mitogen-activated protein kinase (MAPK) cascade and the nuclear factor kappa B (NF-κB) pathway^22^. Interestingly, activation of NF-κB is also the major inducer of IL-6 transcription and release^24^, which might represent a common link between IL-6 and sIL-6R generation. Whether the activation of other TLRs besides TLR4 is also able to induce sIL-6R release, presumably by activation of a metalloprotease, has not been investigated systematically so far.

In the present study, we show that activation of TLR2 on primary human peripheral blood mononuclear cells (PBMCs) and THP-1 cells induces the generation of sIL-6R by ADAM10 and ADAM17. We further identify monocytes as the cellular source and show that TLR2 stimulation also leads to the release of IL-6 from monocytes, which is differentially regulated by the ERK signaling pathway. Our results provide an unknown molecular link between infection and sIL-6R generation in the initiation of an immunological response.

## Results

### sIL-6R levels barely increase after TLR4 activation on human cells or during sepsis

**Figure 1:**
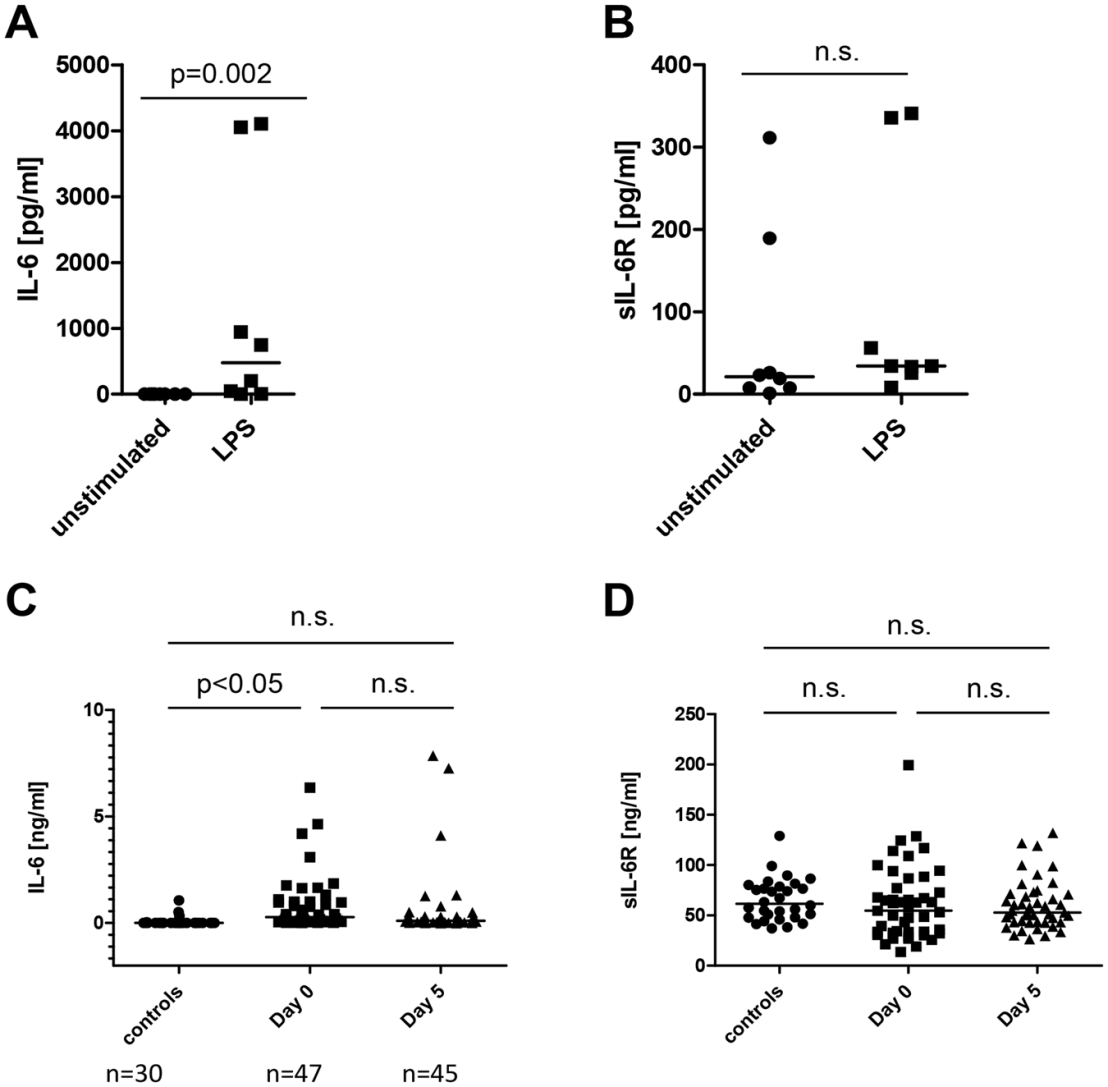
Activation of TLR4 is only a weak inducer of IL-6 trans-signaling. (**A**,**B**) THP-1 cells were incubated with or without the TLR4 activator LPS (5 µg/ml) for 24 h. Supernatants were collected and IL-6 or sIL-6R levels were determined via ELISA. Shown are the individual data points from three independent experiments (n = 8), which were analyzed by Mann-Whitney-U test (n.s.: not significant). (**C**,**D**) Serum samples from sepsis patients at the day of sepsis diagnosis (day 0, n = 47) and 5 days later (day 5, n = 45) were used to determine IL-6 or sIL-6R levels via ELISA. Serum samples from healthy donors (n = 30) were applied as control. Individual data points are shown and the median is indicated by a horizontal line. Data were analyzed by one-way ANOVA followed by Bonferroni’s Multiple Comparison test (n.s.: not significant).

Mechanisms that lead to the generation of sIL-6R *in vivo* remain largely unknown but previous work showed that activation of the TLR4 via injection of LPS into mice stimulates IL-6R cleavage by the metalloprotease ADAM17^19^. We therefore investigated whether this pathway is also relevant in humans and stimulated human PBMCs with 5 µg/ml LPS for 24 h or left them untreated. Because LPS is also a major inducer of IL-6^25^, we first measured the amount of secreted IL-6 in the supernatant of the cells via ELISA. As expected, LPS-treated cells secreted significantly more IL-6 into their supernatant compared to the unstimulated cells (Fig. 1A). Surprisingly, we could not identify such a difference in the amount of sIL-6R (Fig. 1B), suggesting that LPS via TLR4 was not a strong activator of ADAM17 and thus sIL-6R generation in this *in vitro* setting.

In order to substantiate this finding, we analyzed serum samples of a cohort of sepsis patients and compared IL-6 and sIL-6R levels to age- and sex-matched controls^26^. While IL-6 was hardly detectable in healthy controls (0.06 ± 0.04 ng/ml), sepsis patients within the first 24 h of diagnosis (referred to as day 0 here) had significantly increased IL-6 levels (0.84 ± 0.2 ng/ml, Fig. 1C), which declined slightly five days after diagnosis (0.61 ± 0.2 ng/ml). In contrast, we found no difference in sIL-6R levels between healthy controls (65.5 ± 3.8 ng/ml), sepsis patients on day 0 (61.1 ± 5.3 ng/ml) and sepsis patients on day 5 (60.2 ± 3.7 ng/ml, Fig. 1D). In conclusion, these results indicate that LPS is not a strong activator of sIL-6R generation in human cells *in vitro* and in sepsis patients *in vivo*.

### Activation of TLR2 is the strongest inducer of sIL-6R generation among different human TLRs

**Figure 2:**
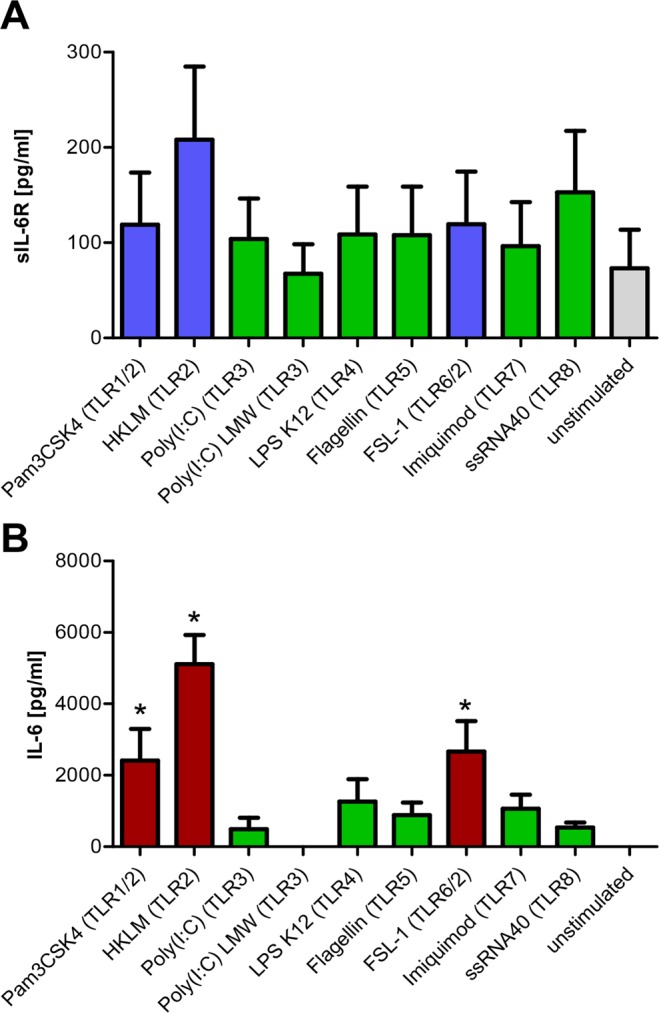
Activation of TLR2 is a potent inducer of IL-6 and sIL-6R release. **(A**,**B**) PBMCs were incubated with different TLR agonists for 24 h as indicated. Supernatants were collected and IL-6 or sIL-6R levels were determined via ELISA. Data shown are the mean ± SEM from four independent experiments, which were analyzed by one-way ANOVA followed by Dunnett’s Multiple Comparison test. Statistical significance compared to the unstimulated cells is indicated with an asterisk.

Because our initial experiments ruled out TLR4 as a major activator of sIL-6R generation, we hypothesized that activation of other TLRs might result in increased sIL-6R generation. In order to systematically address this question, we stimulated PBMCs with a panel of agonists that activate different TLRs: Pam3CSK4 (an activator of TLR1/2), HKLM (TLR2), Poly(I:C)/Poly(I:C) LMW (both TLR3), LPS K12 (TLR4), flagellin (TLR5), FSL-1 (TLR6/2), imiquimod (TLR7), ssRNA (TLR8) or left the cells untreated. Although none of the stimuli resulted in statistically significant increased sIL-6R amounts compared to the unstimulated cells, activation of TLR2 resulted in the highest amounts of sIL-6R (208 ± 77 pg/ml), which is a threefold increase compared to unstimulated cells (73 ± 40 pg/ml, Fig. 2A). Importantly, activation of TLR4 again did not result in a profound increase in sIL-6R. We also quantified IL-6 levels in the same samples via ELISA. As shown in Fig. 2B, activation of the individual TLRs resulted in varying amounts of IL-6. While unstimulated cells secreted no IL-6, all stimuli that activated TLR2 heterodimers resulted in significantly increased IL-6 amounts. Treatment with HKLM, which activates all TLR2 heterodimers, was the strongest inducer of IL-6 (5.1 ± 0.8 ng/ml, Fig. 2B). Thus, activation of TLR2 on PBMCs results in the simultaneous release of IL-6 and sIL-6R.

### Monocytes are the major source of IL-6 and sIL-6R

**Figure 3:**
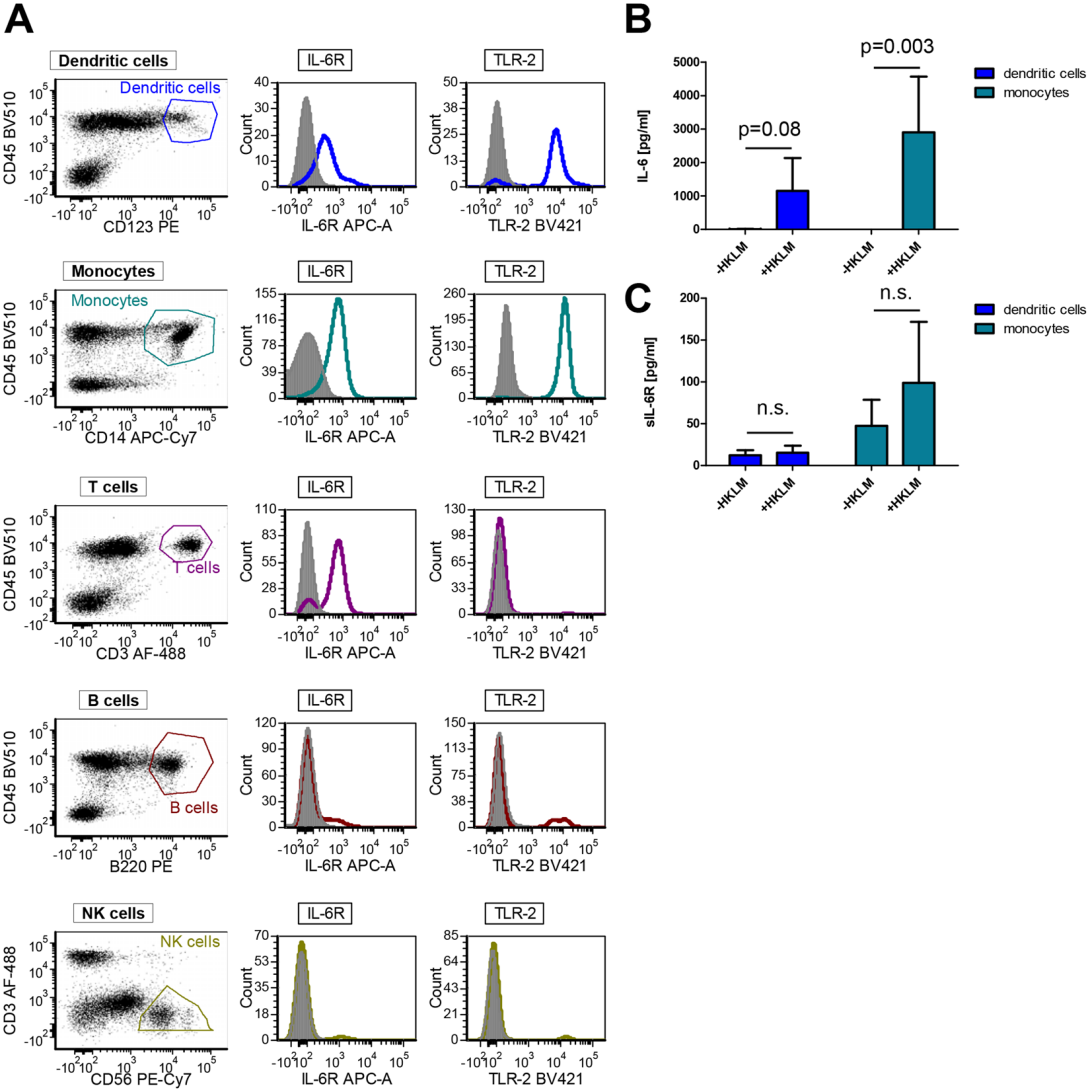
Monocytes and dendritic cells express IL-6R and TLR2 simultaneously but differ in their effects on IL-6 secretion and sIL-6R release. (**A**) PBMCs were isolated from plasma-free blood by a density gradient centrifugation using Ficoll Histopaque. After staining with specific antibodies cells were analyzed by flow cytometry and gated as follows: CD45^+^CD123^+^ dendritic cells, CD45^+^CD14^+^ monocytes, CD45^+^B220^+^ B cells, CD45^+^CD3^+^ T cells and CD45^+^CD56^+^ CD3-negative NK cells. Additionally, surface IL-6R and surface TLR2 were stained and are shown for each cell subset. (**B**,**C**) Monocytes and dendritic cells were isolated from PBMCs using the antibody-based magnetic cell separation. The isolated cells were incubated with the TLR2 agonist HKLM (10^8^ cells/ml) for 24 h. The supernatants were collected and the amount of (**B**) IL-6 and (**C**) sIL-6R was determined via ELISA. Data shown are the mean ± SD from two independent experiments (n = 3). A p value < 0.05 was considered as statistically significant (Mann-Whitney-U test, ns = not significant).

Having found that activation of TLR2 is critical for the generation of both IL-6 and sIL-6R, we sought to identify the subpopulation of PBMCs from which both proteins originate. PBMCs include monocytes, B cells, T cells, NK cells and immature dendritic cells. In order to analyze these cells, we employed a flow cytometry-based strategy and used different cell surface markers for the individual cell populations and co-stained them with antibodies directed against the IL-6R and TLR2. Specifically, we stained PBMCs with an antibody directed against CD45, a marker found on all leukocytes, to distinguish these cells from other cells present in the PBMC suspension. Additionally, CD14 was used to identify monocytes, CD123 for dendritic cells, CD3 for T cells, B220 for B cells and CD56/CD3 for NK cells (Fig. 3A). Using this gating strategy, we distinguished the individual cell populations and co-stained TLR2 and IL-6R on them. As shown in Fig. 3A, gating for CD45^+^CD123^+^ dendritic cells and CD45^+^CD14^+^ monocytes revealed that these were the only two cell populations simultaneously expressing IL-6R and TLR2. We found IL-6R expression on T cells, as it has been reported earlier^27,28^, but could not detect expression of TLR2 here. TLR2 expression on T cells has been shown previously^29^, but this discrepancy is most likely caused by different staining protocols. Both proteins were only marginally detectable on B cells and NK cells, making these two cell types unlikely sources of the sIL-6R.

In order to address the individual capacity of dendritic cells and monocytes to produce IL-6 and sIL-6R, we isolated both cell types from PBMCs via antibody-based magnetic cell separation and stimulated them with the TLR2 agonist HKLM. While unstimulated dendritic cells released no IL-6 into the supernatant, HKLM-treatment induced IL-6 secretion, indicating that stimulation of TLR2 was an effective IL-6 inducer in this cell type (Fig. 3B). When we performed the same experiment with monocytes, HKLM-treatment similarly led to the secretion of IL-6 at even higher amounts, whereas unstimulated monocytes secreted no IL-6 (Fig. 3B). When we analyzed the same supernatants for sIL-6R via ELISA, we found that unstimulated dendritic cells released small amounts of sIL-6R, but this was not increased when the cells were treated with HKLM (Fig. 3C). On monocytes, sIL-6R release was stronger compared to dendritic cells, even without stimulation, and increased when the cells were stimulated with HKLM. In summary, we identify monocytes and to a lesser extent dendritic cells as the cellular sources of IL-6 secretion, while sIL-6R appears to be predominantly produced by monocytes, especially after TLR2 activation.

### Activation of TLR2 induces IL-6R proteolysis by ADAM10 and ADAM17

**Figure 4:**
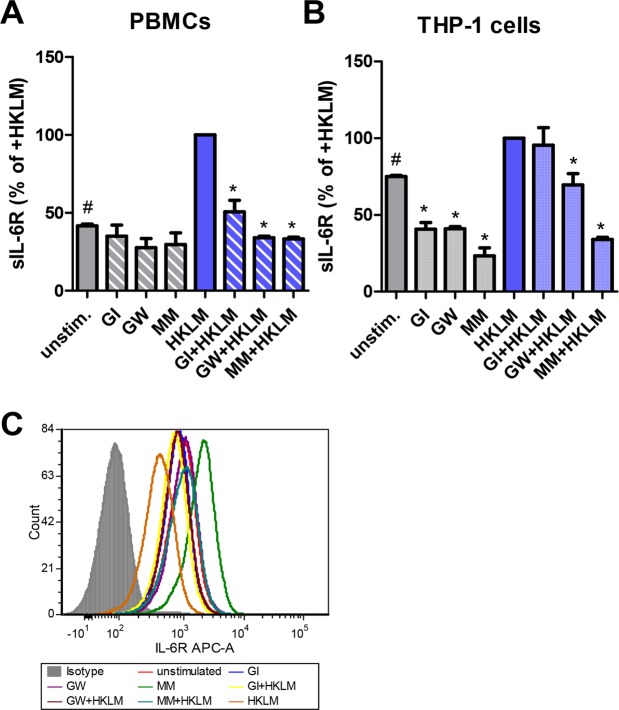
sIL-6R release after TLR2 activation involves proteolysis by ADAM10/17. (**A**) PBMCs were isolated from plasma-free blood and were pre-incubated with protease inhibitors GI, GW or Marimastat (MM) for 30 min. Afterwards, TLR2 was stimulated with HKLM (10^8^ cells/ml) for 24 h where indicated. The supernatants were collected and the amount of sIL-6R was determined via ELISA. Data shown are the mean ± SD from two independent experiments (n = 3). “^#^” indicates a statistical significant difference (p < 0.05, one sample t-test) between unstimulated and HKLM-treated cells and “*” a statistical significant difference (p < 0.01, one-way ANOVA with Dunnett’s Multiple Comparison Test) between HKLM-treated cells and HKLM-treated cells additionally pre-incubated with the three different metalloprotease inhibitors. (**B**) THP-1 cells were resuspended in serum-free DMEM and treated as described in panel A. Data shown are the mean ± SD from three independent experiments. “^#^” indicates a statistical significant difference (p < 0.05, one sample t-test) between unstimulated and HKLM-treated cells and “*” a statistical significant difference (p < 0.01, one-way ANOVA with Dunnett’s Multiple Comparison Test) between either untreated or HKLM-treated cells and the respective cells additionally pre-incubated with the three different metalloprotease inhibitors. (**C**) THP-1 cells were resuspended in serum-free DMEM and 2 samples each were pre-incubated with protease inhibitors GI, GW, MM or DMSO control for 30 min. Afterwards, the cells were stained with antibody against the IL-6R. THP-1 cells were washed, resuspended in 1 ml serum-free DMEM and GI, GW, MM or DMSO were added again. Cells were stimulated for 24 h with HKLM where indicated and remaining surface IL-6R was stained with an APC-conjugated anti-mouse IgG mAb afterwards and analyzed by flow cytometry.

We have previously shown that both ADAM10 and ADAM17 are able to cleave the IL-6R following an appropriate stimulus^11,13–15,18^. In order to analyze whether one or both proteases are responsible for sIL-6R generation after TLR2 activation, we incubated PBMCs with different metalloprotease inhibitors. Neither GI, which specifically inhibits ADAM10, nor GW (specific for ADAM10 and ADAM17) or marimastat (MM, broad spectrum metalloprotease inhibitor) had a strong influence on the basal sIL-6R release from PBMCs, suggesting that rather alternative splicing than limited proteolysis is the responsible mechanism here (Fig. 4A). On the other hand, when we stimulated PBMCs with HKLM, the increase in sIL-6R was blocked by all three metalloprotease inhibitors significantly, pointing to ADAM10 as the responsible protease (Fig. 4A). However, GW and MM were more potent than GI in blocking sIL-6R generation, making a contribution of both ADAM10 and ADAM17 the most likely explanation.

Furthermore, we verified the involvement of ADAM proteases after TLR2 activation in the monocytic cell line THP-1 (Fig. 4B). Here, proteolysis of the IL-6R was blocked by GI, GW and MM in unstimulated cells, confirming a role of ADAM10 in the constitutive release of the IL-6R, as reported earlier^12,13^. In contrast, HKLM-induced sIL-6R generation was blocked by GW and MM, but not GI, suggesting ADAM17 as the responsible protease (Fig. 4B). To further substantiate that the observed effects indeed are caused by proteolysis of the IL-6R and do not involve altered transcription or alternative splicing, we confirmed these findings via flow cytometry. HKLM-treatment of THP-1 cells led to a reduction of IL-6R on the cell surface, which was not the case when the cells were pre-treated with a metalloprotease inhibitor (Fig. 4C). Interestingly, addition of MM to the THP-1 cells without HKLM-treatment increased the amounts of IL-6R on the cell surface, confirming the results in Fig. 4B that constitutive sIL-6R release is caused by a metalloprotease that can be inhibited by MM.

### The Extracellular-signal regulated kinase (ERK) cascade differentially regulates IL-6 and sIL-6R release in PBMCs and monocytes

**Figure 5:**
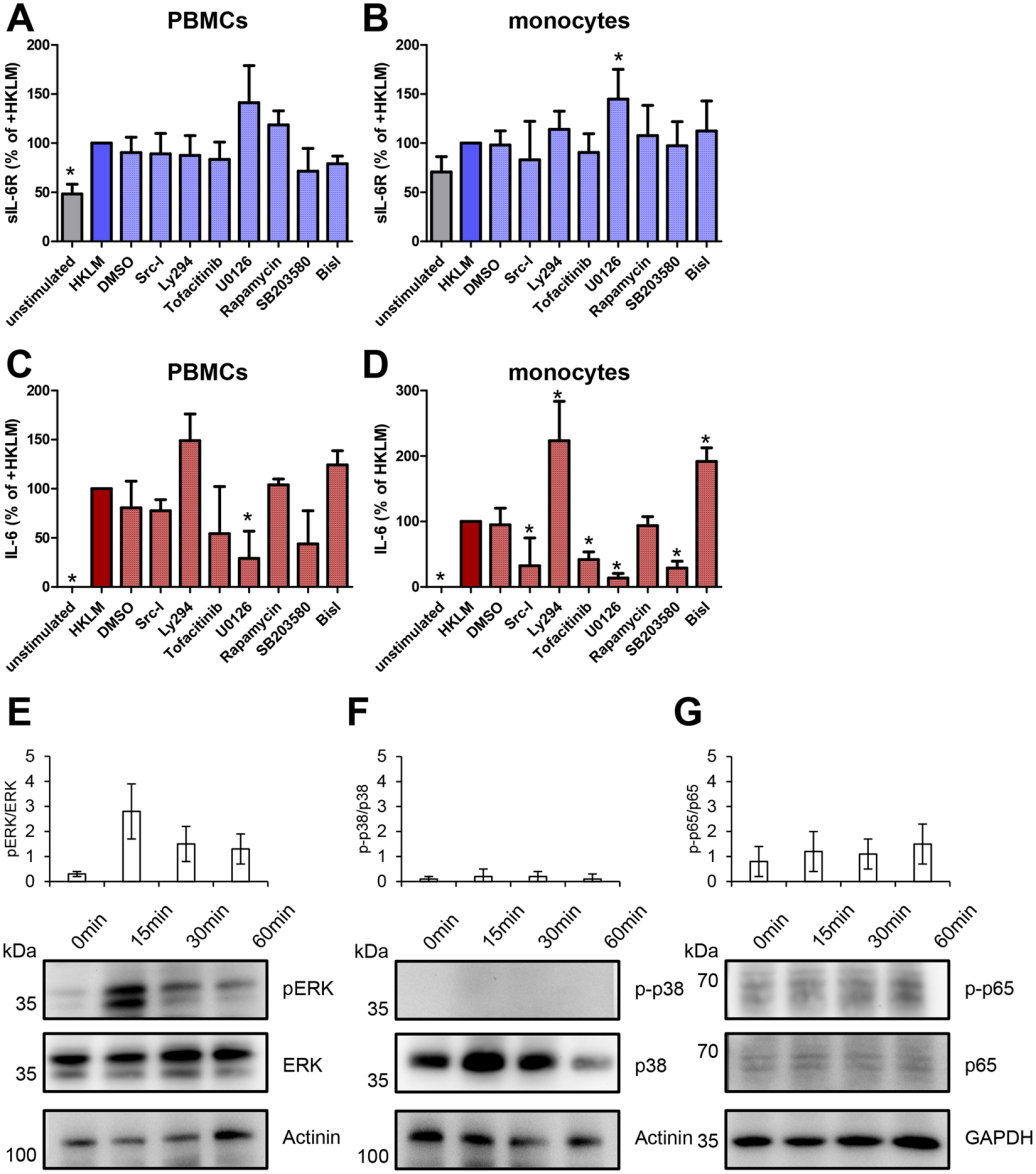
Stimulation of TLR2 results in the activation of Extracellular-signal Regulated Kinase (ERK) cascade which regulates IL-6 and sIL-6R levels differentially. (**A**) PBMCs were isolated from plasma-free blood and were pre-incubated with the signaling pathway inhibitors Src-I (targeting the kinase Src), Ly294 (PI3K), tofacitinib (Jak1/2), U0126 (ERK), rapamycin (mTOR), SB203580 (p38/MAPK) and BisI (targeting PKC) for 90 min. Afterwards, TLR2 agonist HKLM (10^8^ cells/ml) was added and the cells were incubated for further 24 h. The supernatants were collected and the amounts of sIL-6R were determined via ELISA. (**B**) Monocytes were isolated from PBMCs using the antibody-based magnetic cell separation. Stimulation of monocytes and analysis was performed as described in panel A. (**C**,**D**) The experiments were performed as described in panels A and B, but the amounts of IL-6 were determined via ELISA. Data shown are the mean ± SEM from four independent experiments (n = 8). Data were analyzed by one-way ANOVA followed by Dunnett’s Multiple Comparison test. Statistical significance compared to the HKLM-treated cells is indicated. (**E**–**G**) PBMCs were isolated from plasma-free blood, resuspended in serum-free RPMI and were starved for 2 h. The cells were stimulated with the TLR2 agonist HKLM (10^8^ cells/ml) for 0, 15, 30 and 60 min and lysed afterwards. The indicated proteins were analyzed by Western blotting. Quantification is shown in the histograms above the Western blots. Data are representative of three independent experiments.

Having found that ADAM-mediated proteolysis of the IL-6R and IL-6 secretion occur in response to TLR2 activation, we sought to analyze which intracellular pathways are required for these processes. Therefore, we tested a variety of chemical compounds that target these, including Src-I (targeting the kinase Src), Ly294 (PI3K), tofacitinib (Jak1/2), U0126 (ERK), rapamycin (mTOR), SB203580 (p38/MAPK) and BisI (targeting PKC). We again isolated PBMCs, pre-treated the cells for 90 min with the different inhibitors or DMSO as solvent control, and stimulated the cells afterwards with HKLM for 24 h. As shown in Fig. 5A, most inhibitors had no influence on sIL-6R generation, as judged by ELISA measurement. The fact that inhibition of PKC did not reduce the amount of sIL-6R was surprising, because activation of PKC is regarded as the major inducer of ADAM17 activation^30^. Although p38/MAPK has also been described as an important intracellular pathway that leads to activation of ADAM proteases^31,32^, inhibition of p38/MAPK had no effect on sIL-6R generation (Fig. 5A). Interestingly, sIL-6R generation was increased when we applied U0126, an inhibitor of the ERK signaling cascade. We verified our observation in purified human primary monocytes, which confirmed that inhibition of ERK signaling enhanced sIL-6R generation (Fig. 5B).

We used the same supernatants in order to additionally analyze IL-6 generation. As already shown, HKLM-treatment of PBMCs resulted in the secretion of IL-6 from PBMCs, while no IL-6 was detected in unstimulated cells (Fig. 5C). Inhibition of Src, mTOR and PKC had no apparent effect on IL-6 secretion, while blockade of PI3K rather enhanced the IL-6 release. In contrast, IL-6 was decreased when cells were pre-treated with inhibitors of Jak1/2, p38/MAPK and ERK, while only the latter one reached statistical significance (Fig. 5C). We also verified these effects in supernatants from stimulated monocytes, and although the differences were much stronger compared to PBMCs, we basically found the same effects (Fig. 5D).

Having identified ERK as the major pathway affected by our inhibitor screening, we sought to verify that the signaling cascade was indeed activated after TLR2 activation. Several signaling cascades have been described downstream of TLR2, including NF-κB^33^, p38/MAPK^34^ and ERK^35^. Therefore, we stimulated PBMCs with HKLM and harvested cells after 0, 15, 30 and 60 min. Indeed, ERK was strongly phosphorylated after 15 min of TLR2 activation, which declined afterwards in a time-dependent manner (Fig. 5E). We did not detect a phosphorylation of p38 (Fig. 5F) and could only detect a weak phosphorylation of the NF-κB subunit p65 after 60 min of stimulation with HKLM (Fig. 5G). Because inhibition of Src, PI3K, Jak1/2, mTOR and PKC had no effect on sIL-6R generation, these pathways were not further analyzed. Thus, while inhibition of ERK on the one hand led to higher sIL-6R levels in the cell culture supernatant, IL-6 secretion on the other hand was blocked, which suggests that the ERK signaling cascade plays a critical decisive role in controlling IL-6 signaling downstream of TLR2.

## Discussion

The IL-6 signaling cascade has gained remarkable attention as a valuable therapeutic target for the treatment of a wide variety of inflammatory diseases^7^. Consequently, a monoclonal antibody that binds to and thereby blocks the IL-6R is clinically approved in more than 160 countries, e.g. for the treatment of rheumatoid arthritis^5,36^. Notably, IL-6 trans-signaling via the soluble IL-6 receptor is believed to account for the pro-inflammatory properties of IL-6, and the selective inhibition of this signaling axis might therefore represent a superior way of IL-6 blockade^37^. Despite this, the responsible signaling pathways for the generation of the sIL-6R are largely unknown.

Here, we identify TLR2 activation as a novel trigger for IL-6 trans-signaling. Although it is known that TLR signaling and cytokines of the IL-6 family are closely connected^38^, research has concentrated on the activation of TLR4 in this regard. In a mouse model of endotoxemia, injection of LPS into mice resulted in significantly increased sIL-6R serum levels, which was absent in mice deficient for the metalloprotease ADAM17^19^. This finding suggests that ADAM17 acts downstream of TLR4 and proteolytically cleaves the IL-6R. Indeed, we could recently show that the majority of sIL-6R in the human circulation originates from proteolytic cleavage and that the used cleavage site *in vivo* is identical to the cleavage site that ADAM17 uses *in vitro*^11^. One could therefore imagine that a similar molecular pathway is also active in humans and that sIL-6R serum levels rise during sepsis. Indeed, LPS has been shown to induce sIL-6R release from CD14^+^ blood monocytes^39^. However, we could not detect such increase in our patient cohort compared to healthy controls, although IL-6 serum levels were significantly elevated during sepsis, as it has been described previously^2^. These findings suggest that the ADAM17 activation by TLR4 in humans has not the same dominant role that has been described in mice.

TLR2 has not been implicated in sIL-6R generation in humans before. Our data show that sIL-6R release after TLR2 activation was reduced by broadband metalloprotease inhibitors and by inhibitors that specifically targeted ADAM10 and ADAM17. While T cells have been described as a cellular source for the sIL-6R, which is generated by ADAM-mediated proteolysis after T cell activation^27^, they appeared not to be involved in TLR2-induced IL-6R shedding, as we were not able to detect TLR2 expression on them. This is in contrast to previous reports^29^ and most likely caused by different staining protocols. However, both dendritic cells and monocytes could be shown to express surface IL-6R and TLR2 simultaneously, although shedding of the IL-6R in response to TLR2 activation occurred exclusively on monocytes, but not on dendritic cells. In line with this, we have recently described an additional mode of IL-6 signaling, in which IL-6 is loaded onto the IL-6R on the surface of dendritic cells and this cytokine/receptor complex is then trans-presented to cognate T cells, that bear only gp130 but not IL-6R on their plasma membrane, a process that leads to the generation of pathogenic Th17 cells^10^. Also in that study, no significant amounts of free sIL-6R/IL-6 complexes were generated, because T cell activation was not blocked with sgp130Fc, which selectively inhibits IL-6 trans-signaling. The molecular reason why dendritic cells are not able to release substantial amounts of sIL-6R is currently unclear and warrants further investigation. In contrast, IL-6 secretion was induced in a TLR2-dependent manner from both monocytes and dendritic cells. In accordance with our data, sIL-6R generation and therefore IL-6 trans-signaling in a murine model of acute experimental asthma was dependent on TLR2, but not TLR4^40^. It has to be noted, however, that our data rely solely on the activation of TLR2. Further studies should either use cells from TLR2 and TLR4 knock-out mice or use siRNA or CRISPR/Cas9-based editing in human cells to ultimately determine the contribution of these two signaling pathways for the initiation of IL-6 trans-signaling.

Recently, upregulation of TLR2 via STAT3 was described to promote gastric tumorigenesis in a genetic mouse model where gp130 was hyperactivated^41^. Here, genetic deletion of TLR2 reduced expression of pro-inflammatory cytokines, including IL-6. This is in accordance with our finding that TLR2 activation induces secretion of the cytokine IL-6 and suggests that TLR2 is an important signaling receptor upstream of IL-6. Whether inhibition of TLR2 might be an appropriate targeting strategy in IL-6-driven diseases is unclear, however, but should be subject of future studies. Furthermore, it is currently unclear whether and how IL-6 trans-signaling responses might be modulated by the different TLR2 heterodimers that are known.

In summary, we identify in this study TLR2 as a potent inducer of IL-6 trans-signaling, whose activation results in the generation of IL-6 and sIL-6R. Downstream of TLR2, we found that the ERK signaling cascade differentially regulates production of both proteins and might therefore represent a molecular switch that is able to modulate the production of IL-6 and sIL-6R.

## Methods

### Cells and reagents

THP-1 cells were cultured in DMEM high-glucose culture medium (Gibco/ Thermo Fisher Scientific, Waltham, MA, USA) supplemented with 10% fetal bovine serum, penicillin (60 mg/l), and streptomycin (100 mg/l). Isolated PBMCs, monocytes and dendritic cells were cultured in serum-free RPMI1640 (Sigma-Aldrich, St. Louis, MO, USA). All cells were kept at 37 °C and 5% CO_2_ in a standard incubator with a water-saturated atmosphere. The TLR agonists were obtained from Invivogen (San Diego, CA, USA). The two metalloprotease inhibitors GI (selective for ADAM10) and GW (selective for ADAM10 and ADAM17) were synthesized by Iris Biotech (Marktredwitz, Germany) and the broad-spectrum matrix-metalloprotease inhibitor Marimastat was purchased from Sigma-Aldrich, St. Louis, MO, USA, as well as the signaling pathway inhibitors SrcI (SrcI inhibitor), Ly294 (PI3K inhibitor), Tofacitinib (JAK1/2 inhibitor), U0126 monoethanolate (Erk inhibitor), Rapamycin (mTOR inhibitor) and SB203580 (p38/MAPK inhibitor). The PKC inhibitor BisI was purchased from Calbiochem GmbH (Frankfurt, Germany). The following antibodies were used: 4–11 (IL-6R antibody) was expressed and purified in-house, pERK, ERK, phospho-p38, p38, p65, GAPDH and Actinin were purchased from Cell Signaling Technology (Frankfurt/M., Germany), phospho-p65 from biorbyt (Cambridge, United Kingdom). The fluorescently labelled antibodies IL-6R-APC (UV4), CD45-BV510 (HI30), CD14-APC/Cy7 (M5E2), B220-PE (RA3-6B2), CD3-AlexaFlour488 (UCHT1) and CD56-PE/Cy7 (HCD56) as well as matching isotype controls mIgG1-APC (IL-6R; MOPC-21) and mIgG1-BV421 (TLR2; 11G7) were purchased from BioLegend (San Diego, CA, USA), TLR2-BV421 (11G7) was obtained from BD Biosciences. The secondary antibodies mouse-APC, mouse-HRP and rabbit-HRP were obtained from Dianova (Hamburg, Germany).

### Sepsis patients and healthy controls

The study population of the sepsis patients has been described in detail previously^26^. Age- and sex-matched healthy controls for these patients were obtained from the population-based cohorts FoCus^42^ and PopGen^43^.

### Enzyme-linked Immunosorbent assay (ELISA)

For the detection of IL-6 in serum from control patients and sepsis patients and in cell culture supernatants the Human IL-6 ELISA kit from Immunotools (Friesoythe, Germany; Cat.-No. 31670069) was used and for the detection of sIL-6R the DuoSet Human IL-6Rα ELISA kit from R&D systems (Minneapolis, MN, USA; Cat.-No. DY227) was used. Both ELISAs were performed according to the manufacturer’s instructions. For the detection of sIL-6R in sera the samples were diluted 1:100 in blocking buffer, cell culture supernatants were not diluted. For the detection of IL-6 both sera and supernatants were used undiluted. Streptavidin-horseradish peroxidase (R&D Systems, Minneapolis, MN, USA) and the peroxidase substrate BM blue POD (Roche, Mannheim, Germany) were used for the enzymatic reaction which was stopped by addition of 1.8 M sulfuric acid. The absorbance was read at 450 nm on a Tecan rainbow reader (Tecan, Crailsheim, Germany).

### Isolation of PBMCs from plasma-free blood samples

Leukocyte concentrates from healthy adult blood donors were obtained of Transfusion Medicine of the University Hospital Schleswig-Holstein (UKSH) in Kiel, Germany and distributed by the Institute of Immunology, CAU, Kiel, Germany. In accordance with the Declaration of Helsinki, written informed consent was obtained from all blood donors, and the research was approved by the relevant institutional ethic committee of the UKSH (study #D 556/15). For the isolation of peripheral blood mononuclear cells 50 ml (PBMCs) plasma-free blood from blood donations was used which was mixed with 100 ml PBS. 15 ml of Histopaque® 1077 (Sigma-Aldrich, St. Louis, MO, USA) was added to 25 ml of the blood suspension and centrifuged for 20 min at 700 g without the brake of the centrifuge turned on. The upper phase was discarded whereas the interphase which contained the PBMCs was transferred into a new 50 ml tube and mixed with PBS to a total volume of 50 ml. The cells were centrifuged for 10 min at 700 g with the brake of the centrifuge turned on, the supernatant discarded and the pellet resuspended in 50 ml PBS. Centrifugation was performed for 5 min at 170 g and the cells were washed again in 50 ml PBS and centrifuged as before. The cell pellet was resuspended in 50 ml serum-free RPMI1640 (Sigma-Aldrich, St. Louis, MO, USA) and the number of PBMCs was determined using a Neubauer counting chamber.

### Isolation of dendritic cells and monocytes from PBMCs

For the isolation of dendritic cells and monocytes out of PBMCs the method of magnetic cell separation (MACS) was used. Beads, LS columns and the magnetic separator were obtained from Miltenyi Biotec (Bergisch Gladbach, Germany). All reagents, LS columns and the separator were pre-cooled to 4 °C. 4 × 10^8^ PBMCs and CD123-beads were used for the isolation of dendritic cells, for the isolation of monocytes 2 × 10^8^ PBMCs and CD14-beads were used. Isolation was performed according to the manufacturer’s instructions with volumes corrected for the used cell number. For higher purity of the isolated cell types the column was washed with 4 × 3 ml MACS buffer and the procedure of separation was repeated using a new LS column and two washing steps with MACS-buffer. For following experiments the isolated cells were washed once with PBS (300 g, 10 min) and resuspended in serum-free RPMI (Sigma-Aldrich, St. Louis, MO, USA).

### Stimulation of cells with TLR agonists

In order to analyze the effect of different Toll-like receptor agonists on the secretion of IL-6 and sIL-6R into the supernatant, 5 × 10^5^ PBMCs in 1 ml serum-free RPMI (Sigma-Aldrich, St. Louis, MO, USA) were incubated with a panel of Toll-like receptor agonists (Pam3CSK4 (1 µg/ml), Heat Killed *Listeria monocytogenes* (HKLM, 10^8^ cells/ml), Poly(I:C) (25 µg/ml), Poly(I:C) LMW (25 µg/ml), LPS K12 (5 µg/ml), Flagellin (1 µg/ml), FSL-1 (1 µg/ml), Imiquimod (5 µg/ml), ssRNA40 (5 µg/ml) (Human TLR1-9 Agonist kit, Invivogen, San Diego, CA, USA) for 24 h at 37 °C and 5% CO_2_. Afterwards, the supernatant was collected and further analyzed.

### Stimulation of cells with protease inhibitors

To determine the protease involved in the generation of sIL-6R after HKLM addition, 5 × 10^5^ cells in 1 ml serum-free cell culture medium were seeded onto a 24-well plate and 2 samples each were pre-incubated for 30 min at 37 °C with the protease inhibitors GI (specific inhibition of ADAM10; 3 µM), GW (specific inhibition of ADAM10 and ADAM17; 3 µM) or Marimastat (broadband metalloprotease inhibitor; 2 µM). Afterwards, the TLR2 agonist HKLM (10^8^ cells/ml) was added to one well each and the cells were incubated for 24 h at 37 °C and 5% CO_2_. The supernatant was collected and analyzed further.

### Stimulation of cells with different signaling inhibitors

In order to elucidate the signaling pathway involved in the generation of sIL-6R and release of IL-6 after stimulation with HKLM, 5 × 10^5^ cells in 1 ml serum-free cell culture medium were pre-incubated with different signaling pathway inhibitors (SrcI (SrcI inhibitor, 10 µM), Ly294 (PI3K inhibitor, 4 µM), Tofacitinib (JAK1/2 inhibitor, 3 µM), U0126 monoethanolate (Erk inhibitor, 10 µM), Rapamycin (mTOR inhibitor, 500 ng/ml), SB203580 (p38/MAPK inhibitor, 10 µM) and BisI (PKC inhibitor, 500 nM)) for 90 min at 37 °C and 5% CO_2_. Afterwards, the TLR2 agonist HKLM (10^8^ cells/ml) was added and the cells were incubated for 24 h at 37 °C and 5% CO_2_. The supernatant was collected and analyzed further.

### Analysis of IL-6R shedding by flow cytometry

In order to analyze IL-6R shedding, THP-1 cells were first washed in PBS and then resuspended in serum-free DMEM. 1 × 10^6^ cells in 1 ml serum-free DMEM were seeded onto a 24-well plate and 2 samples each were pre-incubated for 30 min at 37 °C with GI, GW, Marimastat or DMSO as control. The cells were washed again with PBS and stained with an antibody against the IL-6R (4–11, 1:100 in FACS buffer [1% BSA/PBS]) for 1 h at 4 °C. After incubation the cells were washed in PBS, resuspended in 1 ml serum-free DMEM and seeded again onto a 24-well plate. GI, GW, Marimastat or DMSO were added and additionally the TLR2 agonist HKLM (10^8^ cells/ml) to one well each. The cells were incubated for 24 h at 37 °C before they were washed in PBS and remaining surface IL-6R was stained with an APC-conjugated anti-mouse mAb (1:100) for 1 h at 4 °C in the dark. Afterwards, the cells were washed again, resuspended in FACS buffer and analyzed by flow cytometry using the BD Biosciences FACS Canto II (Becton-Dickinson, Heidelberg, Germany).

### Analysis of different PBMC subsets by flow cytometry

PBMCs were isolated, washed in FACS buffer (1% BSA in PBS) and resuspended in 50 µl blocking solution (50 µl FACS buffer, 0,5 µl Fc-Block (Human TruStain FcX, BioLegend, San Diego, CA, USA), 0,5 µl human serum). The cells were incubated for 15 min at 4 °C, washed once in FACS buffer and resuspended in antibody solution containing the fluorescently labelled antibodies IL-6R-APC, TLR2-BV421, CD45-BV510, CD14-APC/Cy7, B220-PE, CD3-AlexaFlour488 and CD56-PE/Cy7. Cells were incubated for 60 min at 4 °C in the dark, centrifuged and washed with FACS buffer. To lyse remaining erythrocytes the cell pellet was resuspended in 1x RBC Lysis/Fixation Solution (BioLegend San Diego, CA, USA) diluted in water and incubated for 15 min at room temperature in the dark. The cells were washed again in FACS buffer, resuspended in 200 µl FACS buffer and analyzed by flow cytometry using the BD Biosciences FACS Canto II (Becton-Dickinson, Heidelberg, Germany).

### Activation of signaling pathways and analysis via Western Blot

In order to analyze which pathway is activated after stimulation with the TLR2 agonist HKLM, 5 × 10^6^ monocytes in 5 ml serum-free RPMI for each time point were seeded onto a 6-well plate and starved for 2 h at 37 °C. 10^8^ cells/ml HKLM were added to the cells and incubated at 37 °C for 0, 15, 30 and 60 min before the cells were harvested and lysed in 80 µl lysis buffer (50 mM Tris-HCl, pH 7.5, 150 mM NaCl, 2 mM EDTA, 1 mM NaF, 1 mM Na_3_VO_4_, 1% IGEPAL (NP-40), 1% Triton-X-100, Complete protease inhibitor cocktail). 30 µg total protein of each sample was loaded onto a 10% SDS gel, run for 2 h at 110 V and transferred onto a PVDF membrane (Merck Millipore, Darmstadt, Germany) via semi-dry blot using the Trans-Blot®-TurboTM (Bio-Rad, Hercules, CA, USA) at 1 A and constant 25 V for 40 min. Afterwards the membrane was blocked in 5% milk powder in TBS-T for 1 h at room temperature, washed in TBS-T and incubated with primary antibody over night at 4 °C. The following day the membrane was washed in TBS-T and incubated with HRP-conjugated secondary antibody for 1 h at room temperature. The membrane was washed again in TBS-T and proteins were detected with the ECL Chemocam Imager (Intas Science Imaging, Göttingen, Gemany) using the EMD Millipore™ Immobilon™ Western Chemiluminescent HRP Substrate (Merck Millipore, Darmstadt, Germany).

### Statistical analysis

Statistical analysis was done in GraphPad Prism (GraphPad, La Jolla, CA). Data from multiple groups was analyzed by one-way analysis of variance (ANOVA), two groups were compared by the Mann-Whitney-U test. Corrections for alpha-inflation were done where appropriate. A p-value <0.05 was considered as statistically significant.

## Supplementary information

Supplementary Dataset 1

## Data Availability

No restrictions on the availability of materials or information apply. Requests for data and material should be addressed to the corresponding author (christoph.garbers@med.ovgu.de).
