# Supplementary material for: Activation of Toll-like Receptor 2 (TLR2) induces Interleukin-6 trans-signaling
Source: Sci Rep. 2019 May 13;9:7306. doi: 10.1038/s41598-019-43617-5 (PMC6513869; doi:10.1038/s41598-019-43617-5)

Activation of Toll-like Receptor 2 (TLR2) induces Interleukin-6 trans-signaling

Charlotte M. Flynn[1], Yvonne Garbers[2], Juliane Lokau[1,§], Daniela Wesch[3], Dominik M. Schulte[4], Matthias Laudes[4], Wolfgang Lieb[5], Samadhi Aparicio-Siegmund[1] and Christoph Garbers[1,*,§]

[1]Institute of Biochemistry, Kiel University, Kiel, Germany;
[2]Institute of Psychology, Kiel University, Kiel, Germany;
[3]Institute of Immunology, University Hospital Schleswig-Holstein, Campus Kiel, Kiel, Germany;
[4]Department of Internal Medicine 1, Kiel University, Kiel, Germany;
[5]Institute of Epidemiology, Kiel University, Kiel, Germany

[*]Correspondence to: Dr. Christoph Garbers (christoph.garbers@med.ovgu.de, +49 391 67-15488), Department of Pathology, Otto-von-Guericke-University Magdeburg, Medical Faculty, Magdeburg, Germany; Fax: +49 391 67-15818

[§]present address: Department of Pathology, Otto-von-Guericke-University Magdeburg, Medical Faculty, Magdeburg, Germany

Figure 5E

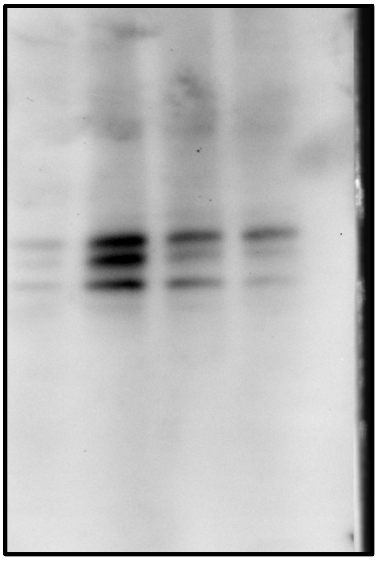

pERK

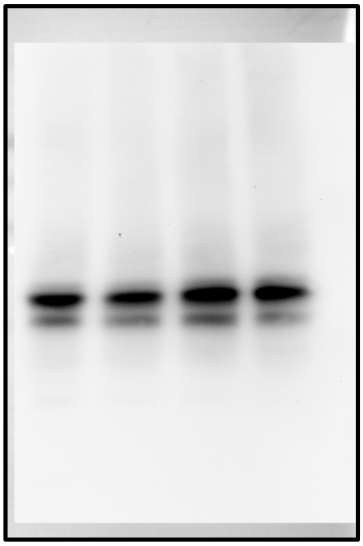

ERK

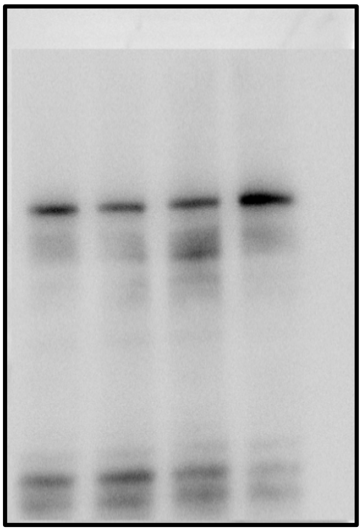

Actinin

Figure 5F

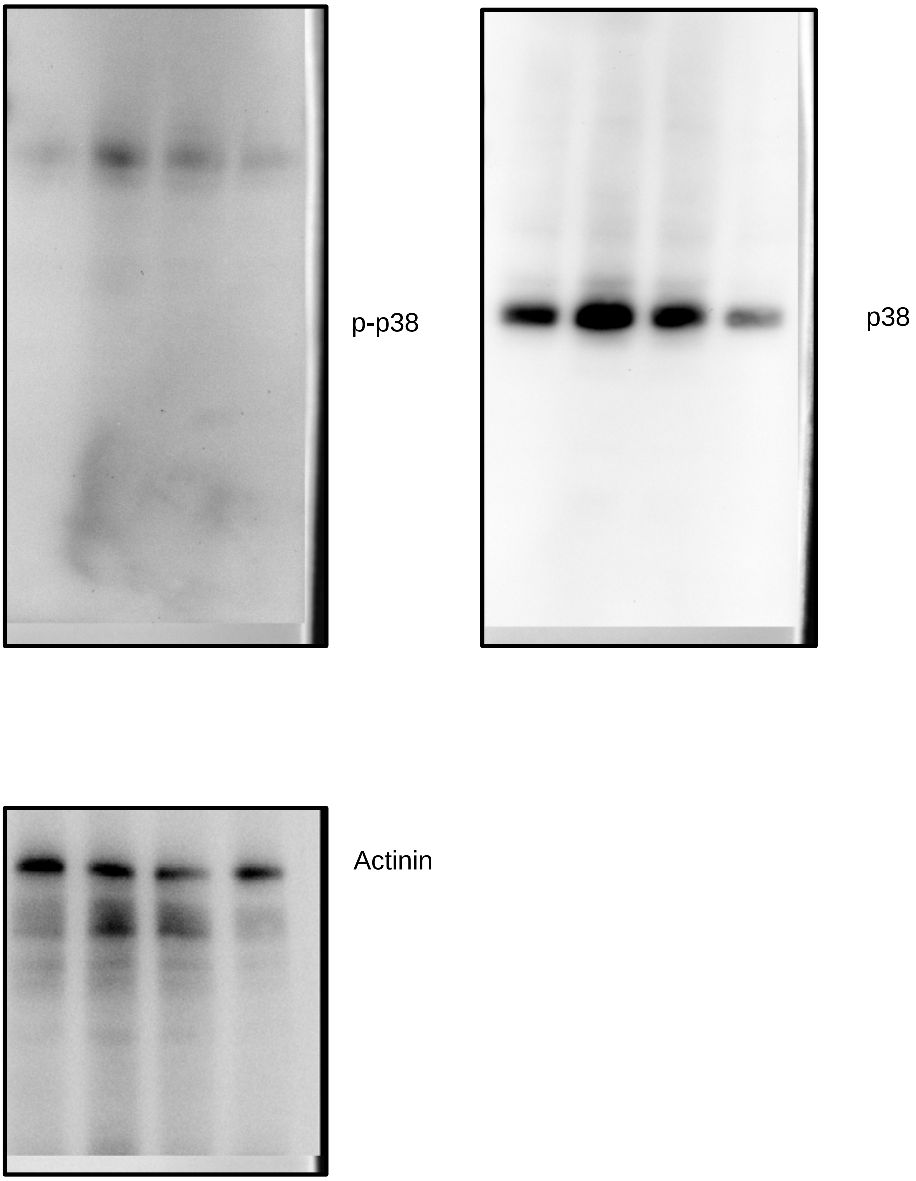

Figure 5G

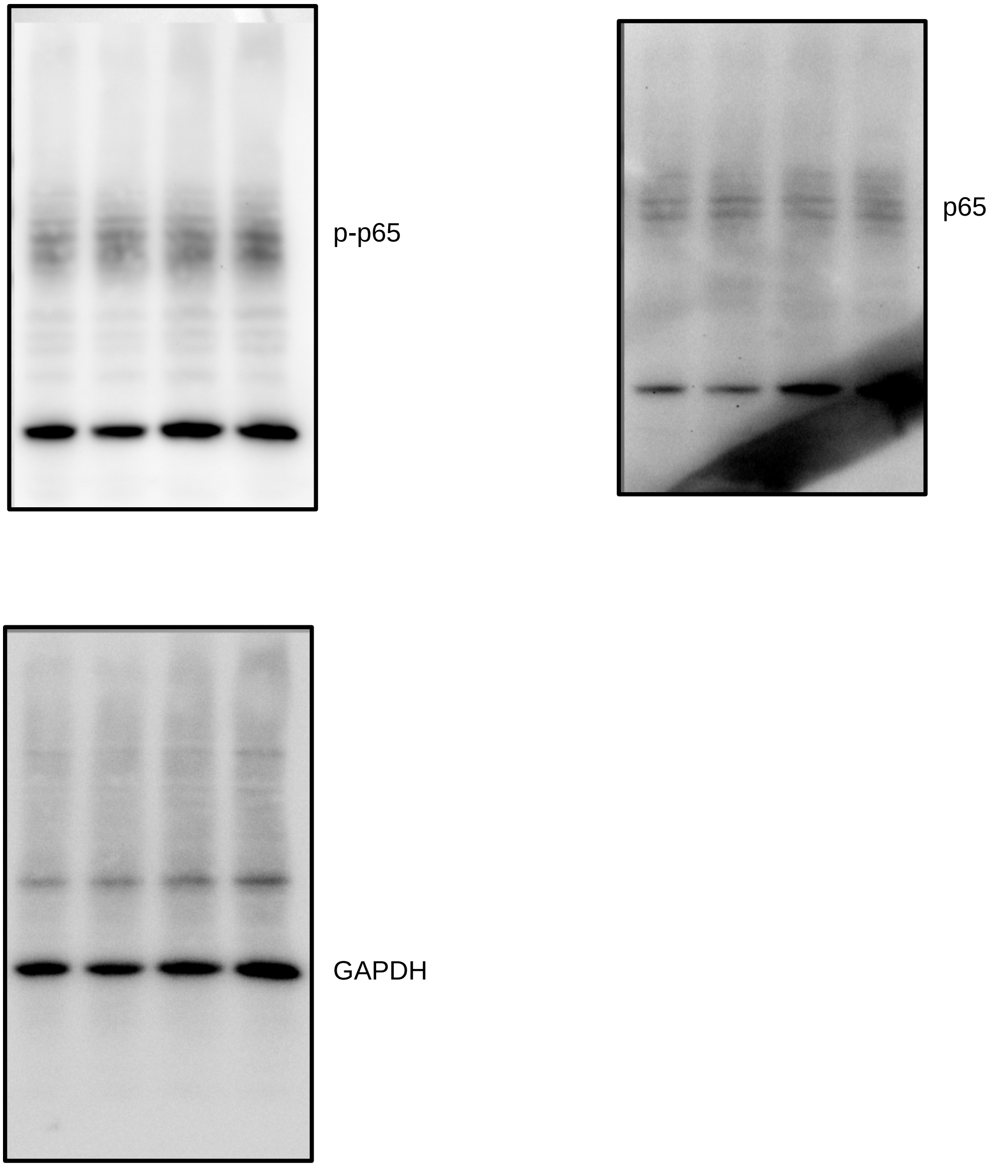

Supplement: Supplementary file 1 — Supplementary Dataset 1 [file 41598_2019_43617_MOESM1_ESM.pdf]
